# Association between the pancreas transplantation and survival of patients with diabetes: A single center experience

**DOI:** 10.1371/journal.pone.0186827

**Published:** 2017-11-02

**Authors:** Ji Yoon Choi, Joo Hee Jung, Sung Shin, Young Hoon Kim, Duck Jong Han

**Affiliations:** Department of Surgery, University of Ulsan College of Medicine and Asan Medical Center, Seoul, Korea; Children's Hospital Boston, UNITED STATES

## Abstract

Pancreas transplantation is considered a therapeutic option for patients with complicated diabetes mellitus. In this study, we compared survival rate between patients on the waiting list for pancreas transplant alone(PTA), simultaneous pancreas–kidney(SPK) transplant, and pancreas after kidney(PAK) transplant and transplant recipients. A total of 503 patients (PTA:n = 116; SPK:n = 303; PAK:n = 84) and 280 PT recipients (PTA:n = 89; SPK:n = 155; PAK:n = 36) were retrospectively analyzed at our center between February 2000 and December 2015; 11.9%(60/503) of the patients on the waiting list and 4.3%(12/280) of the PT recipients died. The overall survival rate was higher in the waiting list group for the first year (99.3% vs. 97.8%), after which it was significantly higher in PT group (p = 0.039). The overall relative risk of all-cause mortality for transplant recipients was 2.145(p = 0.285) for PTA, 0.688(p = 0.735) for PAK, however,0.361 (p = 0.012) for SPK compared with that for the waiting list patients. SPK transplant recipients had considerable higher survival benefits, despite the relatively long waiting period, especially after 1 year. In addition, PTA and PAK can also be considered as a treatment option as patient survival was not poor.

## Introduction

It has been well established that the worldwide prevalence of diabetes mellitus (DM) has steadily increased[1,2]. Moreover, various complications, such as retinopathy and neuropathy, are associated with blood glucose levels and can increase mortality and morbidity rate. In particular, the mortality rate due to cardiovascular disease has increased by 2- to 10-fold in patients with DM compared with that in the general population[1,3,4].

Pancreas transplantation (PT) is considered an acceptable therapeutic option for restoring normoglycemia by supplying sufficient β cells. This was made possible by improvement in both patient and graft survival and postoperative management as well as decrease in postoperative morbidity due to technological progress and development of immunosuppressants[5,6]. However, some studies suggest that intensive insulin therapy can delay the progression of complications[1–6]. The Diabetes Control and Complication Trial demonstrated that improved glycemic control decreased DM-associated microvascular complications[7]. In addition, despite the significant improvement in DM patient survival and restoration of DM complications following PT, drawbacks include perioperative mortality and morbidity as well as the need for immunosuppressants.

Delaying the progression of diabetes complications and increasing life expectancy are the ultimate goals for the treatment of DM. However, it is poorly understood whether PT has a beneficial effect on life expectancy compared with established insulin therapies. There are some large population studies which access the beneficial effect of pancreas transplantation[4,8]. However, they were based on the multicenter registry using United Network for Organ Sharing (UNOS) and International Pancreas Transplant Registry (IPTR) data. So, we planned to collect and analyze the data of our institution as the large volume single center for comparing with the western data.

In the present study, we compared the survival rate of PT recipients with that of patients on the waiting list for PT at a single institution.

## Materials and methods

### Study population

In this study, we enrolled patients who underwent PT and those on the KONOS waiting list. The acceptance onto the waiting list for PT was decided by the KONOS criteria.

**Fig 1:**
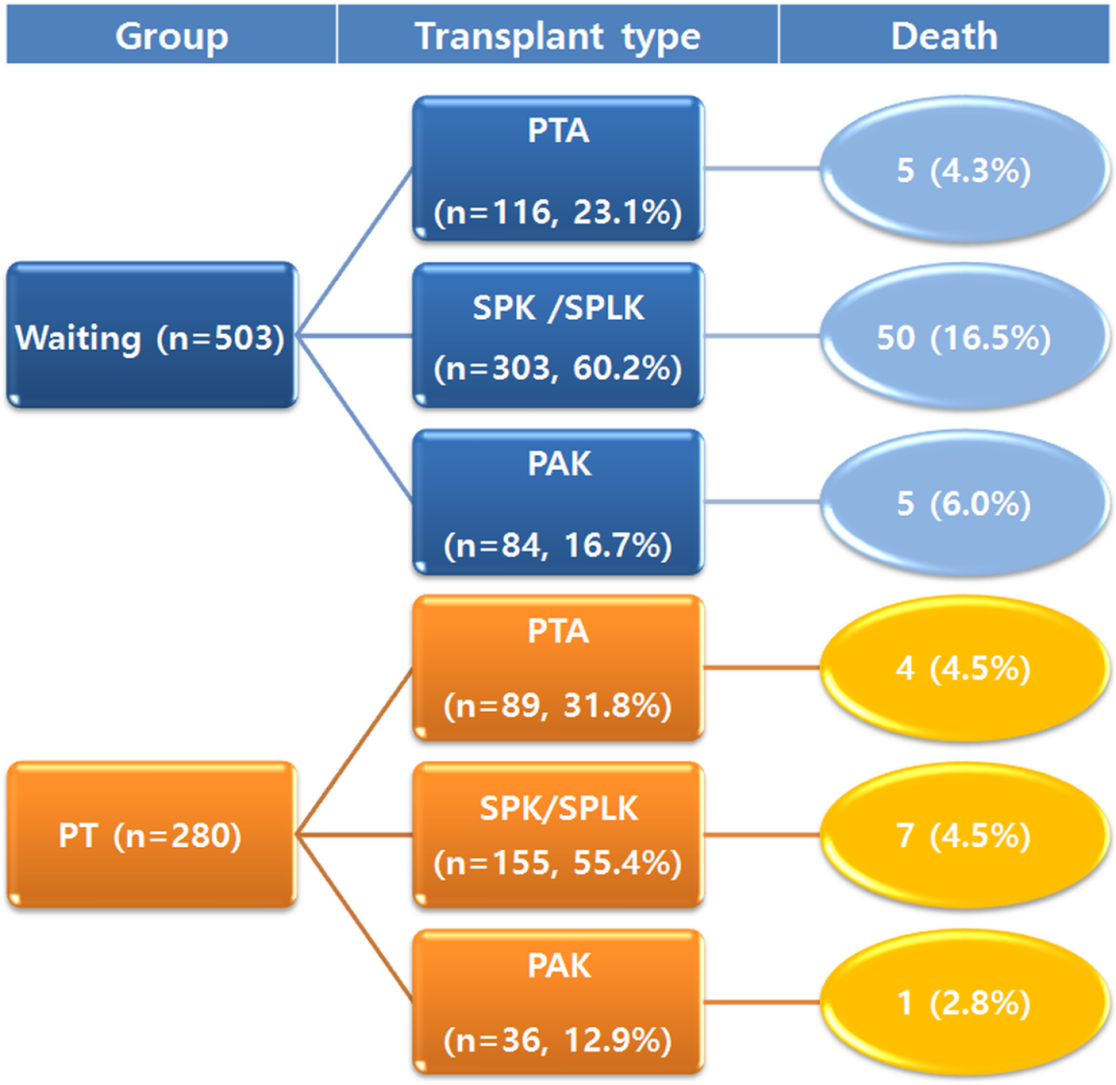
Study population according to the transplant type and mortality.

From February 2000 to December 2015, 890 patients registered our institution for pancreas transplantation. Among them, 504 patients were still on the waiting list for PT and 280 patients underwent PT. We excluded one patient who was listed for multi-organ (other than simultaneous pancreas–kidney) transplantation. Six patients were excluded because we couldn’t follow-up their medical records. As a result, the waiting group consisted of 503 patients and the PT group of 280 patients. In each group, the patients were subdivided according to the transplant type [pancreas transplant alone (PTA), simultaneous pancreas–kidney (SPK) transplant, or pancreas after kidney (PAK) transplant]. In the SPK group, simultaneous pancreas and kidney from a deceased donor and a cadaveric pancreas and kidney from a living donor (SPLK) were included (Fig 1).

### Ethics statement

The study was approved by Institutional Review Board (S2015-1965) of Asan Medical Center. And the need for informed consent was waived due to the retrospective study. The study was conducted in accordance with the 2008 Declaration of Helsinki

### Endpoint

The endpoint was the death of the patients in the two groups. Patient survival was calculated from the time when the patients were diagnosed as diabetes to death or latest follow-up in the waiting list and PT groups. The overall mortality rate and cause were analyzed. To compare patient survival according to the follow-up period, we divided the periods into <1 year and ≥1 year. Patient survival and relative risk of mortality were analyzed according to each period.

The clinical characteristics of the patients at the time when they registered to KONOS for pancreas transplantation were also analyzed according to their group and transplant type. The demographics, duration, and history of DM, amount of insulin, complications of DM, and history of cardiovascular diseases were included.

### Statistical analysis

The categorical variables were analyzed using absolute and relative frequencies with X^2^ test. Quantitative variables were analyzed using mean and standard deviation. Differences between the means were analyzed using a Student’s *t-*test or Mann–Whitney U test. Patient survival was analyzed using Kaplan–Meier estimator and log-rank tests. And the relative risks of patient survival according to their surgery or waiting were analyzed by Cox-regression test. The confounding factors were adjusted. Statistical calculations were performed using SPSS version 21.0 statistical software (SPSS, Chicago, IL) and p < 0.05 was considered statistically significant.

## Results

### Overall mortality of patients

Between February 2000 and December 2015, 116 (23.1%), 303 (60.2%), and 84 (16.7%) patients were on the waiting list for PTA, SPK, and PAK transplants, respectively. During the same period, 89 (n = 31.8%), 155 (55.4%), and 36 (12.9) patients underwent PTA, SPK, and PAK, respectively (Fig 1).

**Fig 2:**
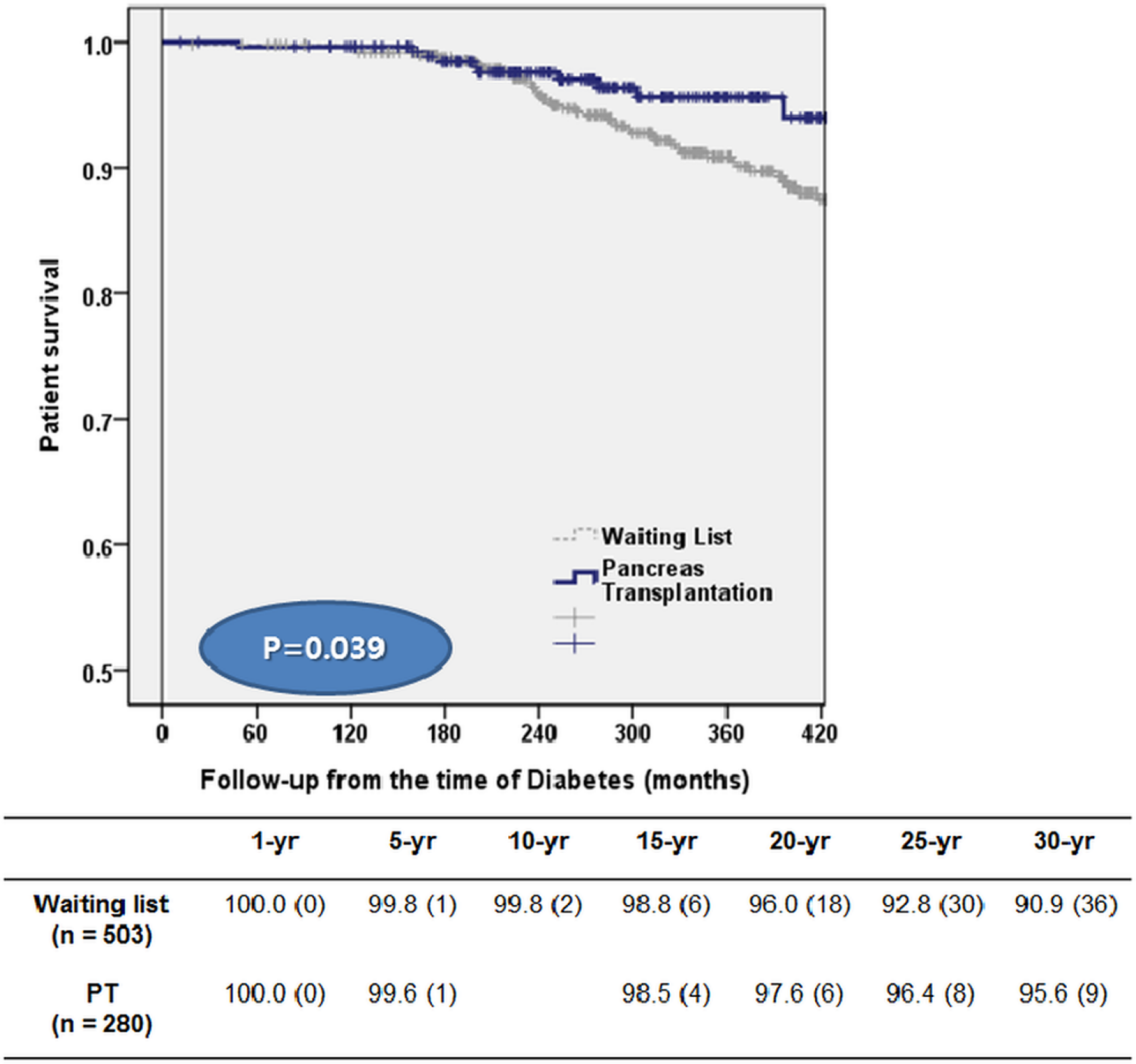
Survival curves comparing the deaths of patients on the waiting list with those after pancreas transplantation.

The demographics and clinical characteristics of the two groups are shown in Table 1. Age and body mass index (BMI) of patients was greater in the waiting group. Detailed data was presented in S1 Data. They also had a significantly greater prevalence of neuropathy, DM foot, hypertension, coronary artery disease, cardiovascular disease, and congestive heart failure. Patients in the PT group showed lower overall mortality rate (4.3%, 12/280than those in the waiting list group (11.9%, 60/503) (p = 0.001) (Fig 2).

**Table 1:** Demographics of patients on the waiting list and recipients of pancreas transplantation.

	Waiting Group (n = 503)	PT group (n = 280)	P-value
**Age (years)**	40.50±11.79 (12–68)	36.21±10.32 (13–65)	0.000
**Gender(Male)**	276 (54.9)	124 (44.3)	0.005
**BMI (kg/m**^**2**^**)**	22.68±3.27 (14.20–33.82)	21.00±2.58 (13.30–29.30)	0.001
**Type 1 DM**	205 (40.8)	224 (80.3)	0.000
**Type 2 DM**	294 (58.4)	55 (19.8)	
**DM onset (age, years)**	24.72±10.47 (3–54)	20.24±9.02 (3–55)	0.000
**DM duration (years)**	15.77±7.42 (4–43)	15.74±7.52 (1–45)	0.953
**Insulin amount (unit)**	34.96±20.65 (0–150)	36.46±21.81 (0–168)	0.360
**Nephropathy**	307/501 (61.3)	190 (67.9)	0.067
**Neuropathy**	141/434 (32.5)	52 (18.6)	0.000
**Retinopathy**	388 (77.1)	198 (70.7)	0.158
**DM foot**	48 (9.5)	10 (3.6)	0.002
**Hypertension**	390 (77.5)	147 (52.7)	0.000
**Coronary artery disease**	58 (11.5)	5 (1.8)	0.000
**Cerebrovascular disease**	18 (3.6)	2 (0.7)	0.015
**Congestive heart failure**	49 (9.7)	0	0.000

The cause of mortality in the waiting rand PT groups was analyzed (Table 2) and was found to be significantly different (p = 0.017). In 29 of the 60 patients on the waiting list, no particular cause could be ascribed, despite attempts to obtain information. Among those recorded, the most common cause of death in the waiting group was cerebrovascular events (7/31, 22.6%) followed by DM complications (6/31, 19.4%). In the PT group, infection (5/12, 41.7%) and others (suicide, hepatic encephalopathy, and pulmonary thromboembolism) (5/12, 41.7%) were the most common causes.

**Table 2:** Comparison of causes of death between waiting and PT group.

	Waiting group (n = 31)	PT group (n = 12)	p-value
**Cardiac disease**	5 (16.1)	1 (8.3)	0.017
**Cerebrovascular disease**	7 (22.6)	0	
**Malignancy**	4 (12.9)	0	
**Infection**	5 (16.1)	5 (41.7)	
Others *	4 (12.9)	5 (41.7)	
**Diabetic complication**	6 (19.4)	0	
**Postoperative bleeding**	-	1 (8.3)	

*Suicide, pulmonary thromboembolism, hepatic encephalopathy

### Clinical characteristics and mortality according to transplant type

**Fig 3:**
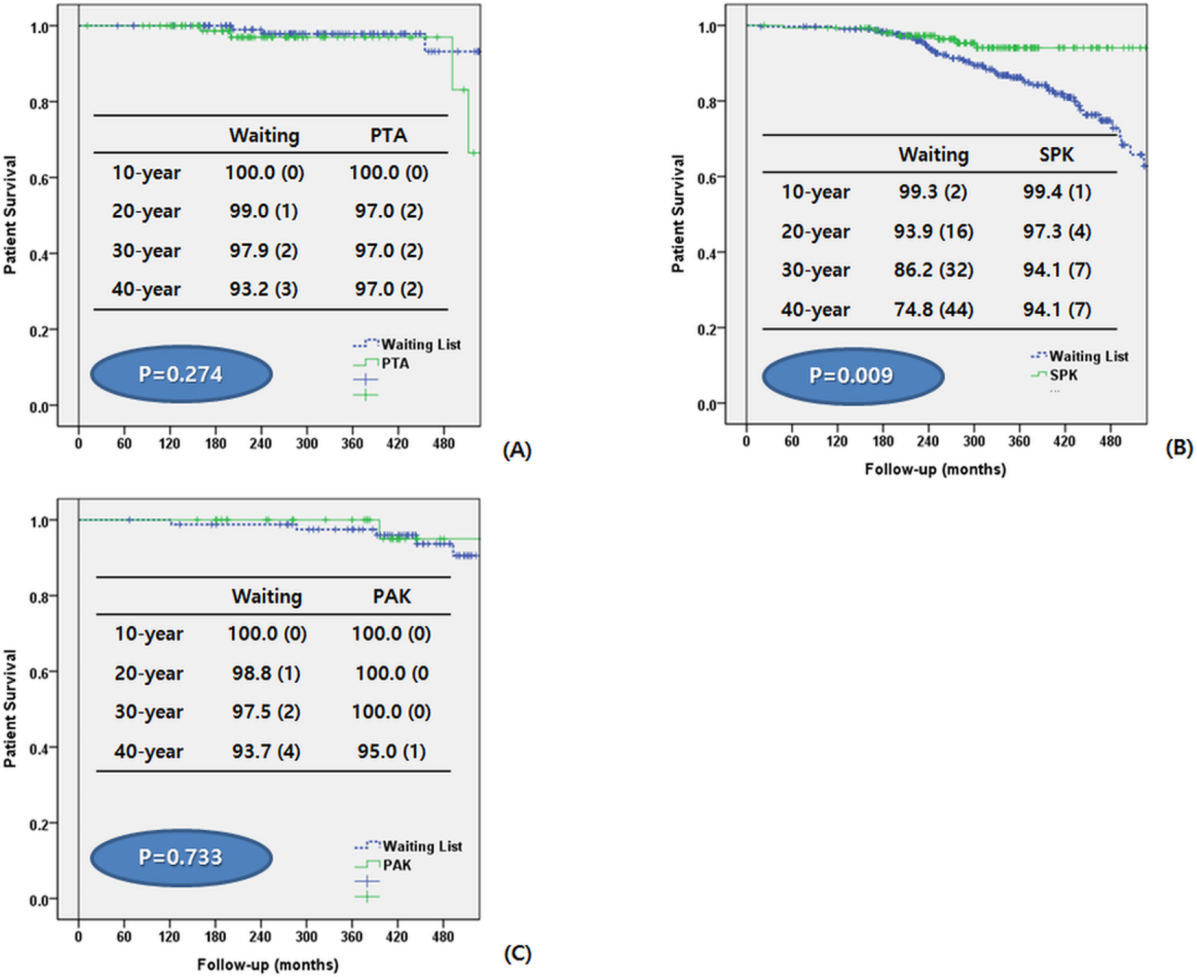
Comparison of patients survival between waiting group and pancreas transplantation group according to transplant type. Follow-up periods were calculated from the diagnosis of DM.(A) Pancreas transplant alone (B) Simultaneous pancreas-kidney transplant (C) Pancreas after kidney transplant.

**Fig 4:**
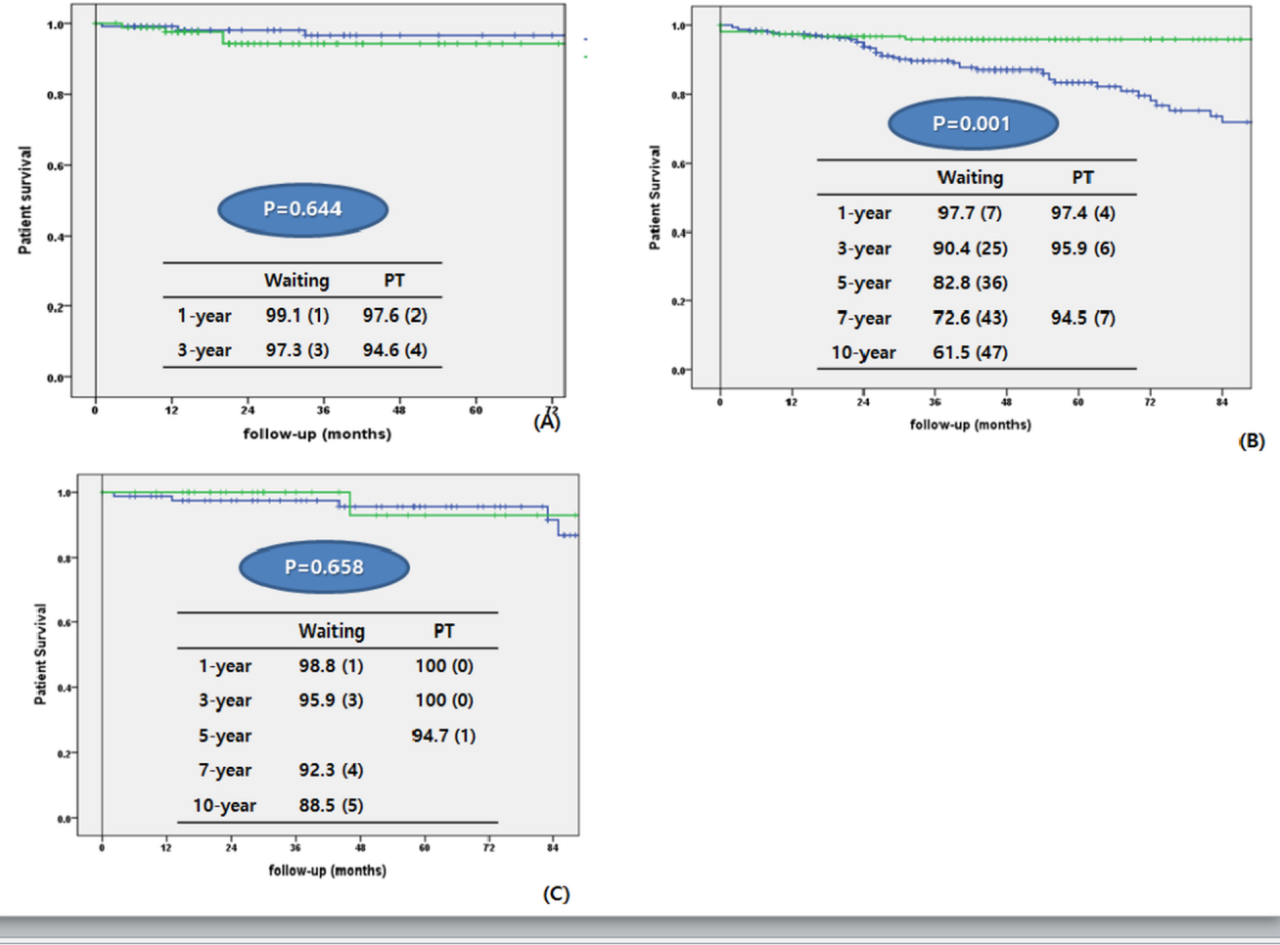
Comparison of patients survival between waiting group and pancreas transplantation group according to transplant type. Follow-up periods were calculated from the registration to KONOS in waiting group and the pancreas transplantation in PT group.(A) Pancreas transplant alone (B) Simultaneous pancreas-kidney transplant (C) Pancreas after kidney transplant.

We compared patients in the waiting group and those in the PT group according to their transplant type (PTA, SPK, and PAK) (Table 3). We also analyzed the patient’s survival from the time of DM diagnosis to the latest follow-up date (Fig 3) and from the time of registration to KONOS and PT to the latest follow-up date (Fig 4)

**Table 3:** Comparison of demographics of patients between waiting group and pancreas transplantation group according to their transplant type. (*p<0.05).

	PTA		SPK		PAK	
	Waiting(n = 116)	PT(n = 89)	Waiting(n = 303)	PT(n = 155)	Waiting(n = 84)	PT(n = 36)
**Age (years)**	32.55±13.19 *	29.42±9.67	41.48±10.10 *	37.97±8.01	47.93±9.03	445.39±10.65
**Gender(Male)**	63 (54.3) *	35 (39.3)	149 (49.2)	42 (46.5)	64 (76.2) *	17 (42.2)
**BMI (kg/m**^**2**^**)**	22.10±3.23 *	21.19±2.68	23.40±12.89 *	20.67±2.44	23.39±2.81 *	21.95±2.68
**Type 1 DM**	69 (59.5) *	84 (94.4)	123 (40.6) *	124 (80)	13 (15.5) *	16 (44.4)
**Type 2 DM**	47 (40.5)	5 (5.6)	180 (59.4) *	31 (20)	71 (84.5) *	20 (55.6)
**DM onset (age, years)**	21.75±10.86 *	18.47±9.96	24.12±9.96	19.63±7.34	31.05±9.16 *	27.22±10.13
**DM duration (years)**	10.74±7.49	10.57±6.68	17.41±6.78	18.10±6.12	16.84±6.41	18.11±8.77
**Insulin amount (unit)**	44.11±20.56	43.23±20.52	30.43±18.64	31.09±21.45	36.42±22.88	42.44±20.67
**Nephropathy**	4 (3.5)	2 (2.2)			3 (3.6) *	34 (94.4)
**Neuropathy**	22 (21.4)	17 (19.1)	93/258 (93) *	30/155 (19.4)	26 (35.6) *	5 (13.9)
**Retinopathy**	55 (47.5)	38 (42.7)	265 (87.5)	128 (82.6)	68 (81.0)	32 (88.9)
**DM foot**	3 (2.6)	1 (1.1)	31 (10.2) *	6 (3.9)	14 (16.7)	3 (8.3)
**Hypertension**	28 (24.1) *	3 (3.4)	283 (93.4) *	118 (76.6)	79 (94.0) *	26 (72.2)
**Coronary artery disease**	2 (1.7)	1 (1.1)	40 (13.2) *	2 (1.3)	16 (19.0)	2 (5.6)
**Cerebrovascular disease**	2 (1.7)	0	10 (3.3) *	0	6 (7.1)	2 (5.6)
**Congestive heart failure**	1 (0.9)	0	41 (13.5) *	0	7 (8.3)	0

For PTA, the mean age of the waiting group was significantly older than that of the PT group (32.55 ± 13.19 vs. 29.42 ± 9.67; p = 0.051). There was more male patients in the waiting group (54.3%, vs. 39.3%; p = 0.033), and BMI and age at DM onset were also higher (p = 0.039 and p = 0.028, respectively). The overall patient survival was not significantly different between the two groups (p = 0.644) (Fig 3A).

For SPK, patient’s age, age at DM onset, BMI, prevalence of neuropathy, DM foot, hypertension, coronary artery disease, and cerebrovascular accidents were significantly higher in the waiting group. Patient survival rate was higher in the waiting group for the first year, after which it was significantly higher in the PT group (p = 0.001) (Fig 3B).

For PAK, age at DM onset, BMI, prevalence of neuropathy and hypertension, and rate of type II DM were significantly higher in the waiting group. Patient survival did not significantly differ between the two groups (p = 0.658) (Fig 3C).

### Relative risk of mortality

**Fig 5:**
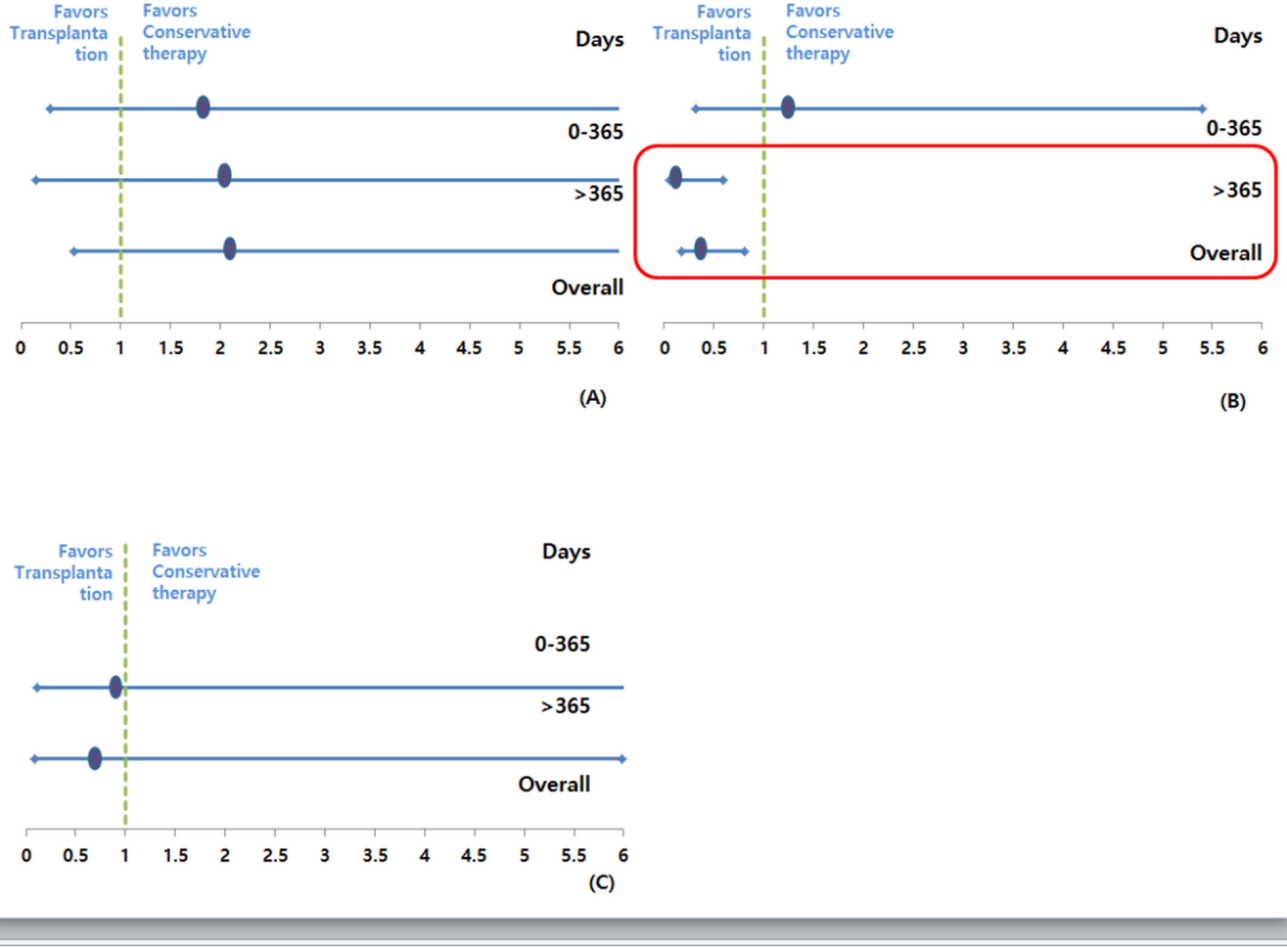
Relative risk of mortality according to transplant type. (A) Pancreas transplant alone (B) Simultaneous pancreas-kidney transplant (C) Pancreas after kidney transplant.

The relative risk of death among the patients on the waiting list was compared with that among transplant recipients for equal follow-up periods (<1 year and ≥1 year) for the same procedure (Fig 5).

For PTA, the overall relative risk of death following transplantation was 2.145 [95% confidence interval (CI): 0.531–8.684; p = 0.285). Until the first year, PTA recipients had a 1.86-fold higher risk of death than patients on the waiting list for the same period (95% CI: 0.146–23.72; p = 0.632). After the first year, they had a relative risk of 2.069 (95% CI: 0.289–14.832; p = 0.049); however, this association was not statistically significant.

SPK recipients exhibited a relative risk of 0.361 (95% CI: 0.163–0.802; p = 0.012) for the follow-up period. For the first year after transplantation, SPK recipients had a 1.306-fold higher risk of death compared with those on the waiting list; however, this was not statistically significant (95% CI: 0.316–5.398; p = 0.182). After the first year, the relative risk was 0.183 (95% CI: 0.057–0.594; p = 0.005).

For PAK recipients, the overall relative risk of death following transplantation was 0.688 (95% CI: 0.079–5.981; p = 0.735). For the first year, there were no deaths in the PT group. After the first year, PAK had a relative risk of 0.949 (95% CI: 0.105–8.611; p = 0.963); however, this was not statistically significant.

## Discussion

Since PT was introduced in 1966 at the University of Minnesota, it has been considered an acceptable treatment based on the improvement in surgical techniques, immunosuppressants, and graft outcomes[5,6]. At our institution, there has been a steady increase in the annual number of PTs performed (≥30 cases since 2012). And we are extending the selection criteria either patients with type I DM or patients with type II DM. However, according to the International Pancreas Transplant Registry data, the number of PTs has steadily decreased over the past decade, with fewer than 1000 cases performed in 2014[7,9]. This trend is more significant for solitary PT, and only 11 centers in the United States performed PT for 20 cases[10,11]. The reasons for the declining number of PT in the United States are multifactorial, including the lack of a primary referral source or acceptance as a primary therapy for DM. On the other hand, Gruessner et al.[8] showed the result in contrast to the result of Venstrom et al.[4]. Until 1 year, PTA didn’t show higher hazard ratio comparing with waiting group. However, for post-transplant day 365 on, PTA showed lower hazard ratio (0.15, 95% CI, 0.08–0.29, p = 0.0001), significantly. Therefore, it is difficult to judge the improvement in long-term patient survival after PT.

Thus, we attempted to analyze and compare the survival rate between patients with DM on the waiting list and PT recipients. Van Dillen et al.[3] previously demonstrated that mortality rate of patients in the waiting list was 30% and that of patients in the PT group was 9% (p = 0.001). In addition, the 1-year mortality rate was 4% for the PT group compared with 13% for the waiting list group (p < 0.001). Venstrom et al.[4] reported that patients waiting for PTA and PT recipients had a 1-year survival rate of 97.6% and 97.1% and a 4-year survival rate of 92.1% and 88.1%, respectively. These reports demonstrated that PT is associated with a relatively low mortality rate during the early postoperative periods as well as long-term survival. When we compared our overall mortality rate with those of other reports, the rates of our two groups were relatively low, with 4.3% in the PT group and 11.9% in the waiting group. This is similar to the patient mortality associated with DM complications (5%–10%)^4^. For the first year, patient survival of the waiting group was higher than that of the PT group. However, after the first year, the patient survival of the PT group was higher.

In this study, we included SPLK group in SPK group for analyzing patient survival. In general, SPLK and SPK are different procedure. In the surgical procedure, SPLK are from two different donors and have a shorter waiting time relatively because they registered on waiting list of solitary pancreas transplantation (PTA or PAK). However, SPK received the organ from one donor and longer waiting time (average 5–6 years in Korea). However, we focused our primary outcome, patient survival. We focused on the patient survival from the time of DM diagnosis. We thought that SPLK and SPK can be considered the same group in that the patients have DM with ESRD as underlying disease and DM with ESRD is corrected after transplantation. Before the transplantation, they had to control DM and do renal replacement therapy such as hemodialysis or peritoneal dialysis. During these periods, DM and its various complications can progress and effect on the patient survival. After transplantation, they don’t need to control DM using exogenous insulin do HD or PD. However, the patients who are on the waiting list have to receive the treatment continuously, and during this period, the disease can progress more and increase the mortality of these patients. For this reason, we considered that the two surgeries (SPLK and SPK) showed the same course of death progression in the disease progression of DM and ESRD patients, and classified into the same group.

The causes of death in each group were significantly different (Table 2). In the waiting list group, cardiovascular disease, cerebrovascular disease, and DM complications were the major causes of mortality; however, infection was the primary cause in the PT group. There was only one death due to surgical complications (bleeding). For the baseline characteristics of the two groups, the patients in the waiting list group had a higher rate of hypertension, ischemic heart disease, cerebrovascular disease, and congestive heart failure than those in the PT group (Table 1).

When we analyzed each group according to their transplant type, we were able to identify that these characteristics were accentuated for SPK. The relative risk associated with SPK was also higher in the PT group than in the waiting group over time. This is reflective of cardiovascular or other complications associated with DM functioning as a major cause of increasing the mortality rate of DM patients. Moreover, the need for life-long immunosuppression can increase the mortality rate following transplantation due to secondary complications (e.g., severe infection) despite improvements in operative techniques and graft outcome. In both PTA and PAK, our data revealed a lower mortality rate in the PT group than in the waiting group. This low mortality rate reveals the survival benefits of PT in patients with DM and normal renal function, and these findings are comparable with those of previous reports[3,4,7,8]. DM patients with renal dysfunction waiting for SPK have a high annual mortality rate, whereas patients waiting for PTA or PAK with preserved renal function have a much better prognosis. This is consistent with the study by Wolfe et al.[12], in which the independent survival benefit was associated with solitary kidney transplant, as well as with the study by Venstrom et al.[4], in which the survival rate of patients waiting for pancreas alone was obviously higher than that of those with renal failure waiting for SPK.

In Venstrom et al.’s study[4], PTA was associated with a 1.57-fold higher relative risk (p = 0.06) of mortality, and PAK showed a 1.42-fold higher relative risk (p = 0.03) of mortality. Moreover, they presented that the survival rate following a PTA was significantly worse compared with that of waiting list patients receiving conventional therapy. However, Gruessner et al.[8] already showed the results in contrast to the study by Venstrom et al.[4]. And our data demonstrated that the relative risk of mortality was not significantly different between the waiting list and PT groups. In addition, only one patient died in the PTA group at our center. Therefore, we suggest that PAK and PTA can be considered an effective treatment modality for DM patients with preserved renal function. That’s because PTA group didn’t show any significant incensement of relative risk for patient survival compared with waiting group.

But, there are some points that we need to caution for our results. Our data showed that patient survival for SPK and PAK are nearly similar (94.1% vs. 95%, 40-year patient survival from the time of DM diagnosis, and 94.5% vs. 94.7%, 7-year patient survival from the registration to KONOS, respectively). And, in PAK, patient survival was not significantly different between patients on waiting list and patients underwent PAK. However, the numbers of patients who listed to waiting list for PAK and underwent PAK are relatively small and it can be one of the limitations for presenting the patient survival correctly. When we reviewed the waiting periods from listing to surgery, solitary pancreas transplantation is performed with 1 year on the average, and many data showed that PAK tend to perform quicker than SPK and better patient survival compared those of patients with diabetes with renal dysfunction. Therefore, for more accurate comparison, the large number of patients(especially in PAK) should be enrolled and other confounding factors should be also considered carefully.

This study has several limitations. It was a single center retrospective analysis and we collected data from 2000; thus, any changes and improvements in immunosuppressants might have an effect on the postoperative mortality regardless of DM. However, our institution is a large volume center which is performed PT and the number of PT is continuously increasing annually. So, we tried to analyze and evaluate the benefit or disadvantage of PT in a single center population.

## Conclusion

Our data suggest that SPK can offer considerable survival benefits despite the associated surgical or immunological complications, particularly in patients with obvious renal impairment. In addition, PTA and PAK can also be considered as a treatment option as patient survival was not poor. However, if renal function is preserved, greater caution should be taken for selecting and determining PT candidates. The actual risks, benefits and perioperative mortality, and morbidity associated with PT and other medical treatments deserve careful consideration.

## Supporting information

S1 Data Data of 504 patients who were still on the waiting list for PT and 280 patients who underwent PT. (XLS)
